# DNA Metabolism in Balance: Rapid Loss of a RecA-Based Hyperrec Phenotype

**DOI:** 10.1371/journal.pone.0154137

**Published:** 2016-04-28

**Authors:** Irina V. Bakhlanova, Alexandra V. Dudkina, Elizabeth A. Wood, Vladislav A. Lanzov, Michael M. Cox, Dmitry M. Baitin

**Affiliations:** 1 Petersburg Nuclear Physics Institute, NRC Kurchatov Institute, Gatchina, 188300, Russia; 2 Peter the Great St. Petersburg Polytechnic University, Saint-Petersburg, 195251, Russia; 3 Department of Biochemistry, University of Wisconsin-Madison, Madison, Wisconsin, 53706–1544, United States of America; Florida International University Bimolecular Sciences Institute, UNITED STATES

## Abstract

The RecA recombinase of *Escherichia coli* has not evolved to optimally promote DNA pairing and strand exchange, the key processes of recombinational DNA repair. Instead, the recombinase function of RecA protein represents an evolutionary compromise between necessary levels of recombinational DNA repair and the potentially deleterious consequences of RecA functionality. A RecA variant, RecA D112R, promotes conjugational recombination at substantially enhanced levels. However, expression of the D112R RecA protein in *E*. *coli* results in a reduction in cell growth rates. This report documents the consequences of the substantial selective pressure associated with the RecA-mediated hyperrec phenotype. With continuous growth, the deleterious effects of RecA D112R, along with the observed enhancements in conjugational recombination, are lost over the course of 70 cell generations. The suppression reflects a decline in RecA D112R expression, associated primarily with a deletion in the gene promoter or chromosomal mutations that decrease plasmid copy number. The deleterious effects of RecA D112R on cell growth can also be negated by over-expression of the RecX protein from *Neisseria gonorrhoeae*. The effects of the RecX proteins *in vivo* parallel the effects of the same proteins on RecA D112R filaments in vitro. The results indicate that the toxicity of RecA D112R is due to its persistent binding to duplex genomic DNA, creating barriers for other processes in DNA metabolism. A substantial selective pressure is generated to suppress the resulting barrier to growth.

## Introduction

DNA metabolism is a set of seemingly distinct processes that are tightly interlinked. The genome must be protected, replicated, expressed, organized, and segregated. All of the processes of DNA metabolism must share the same chromosomal substrate. Spontaneous DNA lesions are ubiquitous, hundreds of thousands appearing daily in a typical human cell, several thousand in each cell within an aerobic bacterial culture [1–3]. The nucleotide excision repair, base excision repair, mismatch repair, and other repair operations that counter these insults typically leave a transient break in the DNA strand undergoing repair. If a replication fork appears before the break is sealed, the fork collapses [4]. The resulting double strand break is perhaps the most dangerous of all DNA damage events [5, 6].

Homologous genetic recombination, or more appropriately recombinational DNA repair, evolved to repair double strand breaks [7–12]. Recombination systems ensure DNA replication success in all cells, facilitating reconstitution of the replication forks that have collapsed upon encounters with those transient template strand discontinuities [7–12]. The capacity to repair double strand breaks was a likely prerequisite to the evolution of large genomes. The enzymatic capabilities inherent to recombinational DNA repair of double strand breaks have been re-purposed by evolution to permit recombination in the context of eukaryotic meiosis, bacterial conjugation, and a host of other functions [13].

The enzymes at the center of recombinational DNA repair systems are the RecA family recombinases. These include the RecA protein of bacteria, the RadA protein of archaea, and the Rad52 and Dmc1 proteins of eukaryotes [14–18]. These proteins function as filaments typically formed on single-stranded DNA (ssDNA). The nucleoprotein recombinase filaments align the bound ssDNA with complementary sequences in an intact duplex DNA, and then promote DNA pairing and strand exchange. Additional enzymes resolve the branched DNA structures created by recombinases. The end product of recombinational DNA repair of a a disintegrated replication fork is a reconstituted fork structure, after replication collapses upon an encounter with a template strand discontinuity,

Whereas recombinational repair is a critical cellular asset, (spurious) homologous recombination is not and in fact can be detrimental. There are at least three ways that recombination systems could harm a cell. First, promiscuous recombination involving repeated chromosomal sequences could re-order or eliminate genes, creating genomic chaos. Second, the nucleoprotein filaments formed by RecA family recombinases are potentially the largest structures that assemble on a bacterial chromosome, and could represent formidable barriers to replication or transcription if not removed from the DNA. Third, unresolved recombination intermediates in DNA could cause genome instability, imparting a different kind of barrier to DNA metabolism and/or chromosomal segregation. In bacteria, recombinase binding to DNA can have additional deleterious effects via the potential induction of the SOS response and its associated halt in cell division and mutagenesis [19].

For these reasons, cellular recombination systems are subject to multiple layers of regulation [20–25]. Here, we focus on bacteria and the RecA recombinase. RecA is expressed only at levels appropriate to the metabolic situation. RecA access to DNA is proscribed under normal circumstances by a very limited capacity to nucleate filament formation on duplex DNA or on ssDNA that is bound by SSB [18, 26–33]. A C-terminal autoregulatory flap in RecA mediates the block to RecA filament nucleation on these DNA substrates [34–37]. The RecA binding protein PsiB sequesters RecA protein under some conditions [38, 39]. Multiple regulatory proteins (RecFOR, DprA, and RecBCD) direct RecA filament formation on SSB-coated single-stranded DNA when required and appropriate [20, 27, 30–33, 40–48]. The regulatory protein DinI modulates RecA filament stability and function after assembly [49–51]. When RecA function is no longer needed, RecA filament removal from the DNA is ensured by additional bacterial regulatory proteins such as RecX protein and DinD protein [50, 52–58], as well as the UvrD, Rep, and PcrA helicases [59–65]. Genetic modulation or ablation of these different controls often has deleterious consequences for the cell [20, 60, 61, 66–70].

Another layer of control on recombination systems has been imposed by evolution. RecA family recombinases have not evolved to promote DNA pairing and strand exchange optimally. Instead, RecA functionality represents an evolutionary compromise that reflects a balance between the critical functions and deleterious consequences of recombination. This balance may be subtly different in each bacterial species. Substantial increases in RecA function can be obtained via mutation [71, 72]. The RecA D112R variant is perhaps the best example of functional enhancement to date, substantially increasing the efficiency of conjugational recombination [71].

The RecA D112R mutant protein was originally isolated as part of an investigation of amino acid residues at the subunit-subunit interface of RecA protein [73]. Additional mutations at codon 112 arose in a study of RecA mutant proteins that were inhibited less by high concentrations of the UmuD and UmuC proteins [74, 75]. In vitro, and relative to wild type RecA, RecA D112R exhibits improvements in loading onto SSB-coated ssDNA, in resistance to inhibitors such as RecX and PsiB, and in pairing homologous DNAs [71]. The enhanced functionality of the D112R mutant protein and other RecA variants gives rise to a broader question. If substantial improvements in RecA functionality are possible, why have they not appeared during evolution? The answer must lie in the potentially deleterious consequences of enhanced RecA recombinase function, which we explore in this report.

## Materials and Methods

### Strains and plasmids

Note: for all protein names, Ec = *Escherichia coli* and Ng = *Neisseria gonorrhoeae*. Donor KL227 (HfrP4x *metB*) and recipients: AB1157 (*thr-1 leuB6 ara14 proA2 hisG4 argE3 thi-1 supE44 rpsL31*) and recombination deficient JC10289 (as AB1157 but *Δ[recA-srlR306]*::*Tn10 = ΔrecA306*) were from A.J. Clark’s collection. An additional ∆araBAD mutation, deleting the entire operon and its control region, was introduced into AB1157 and its derivatives. A plasmid with amino acid substitution D112R in the interface of subunit interactions in the EcRecA filament (pD112R) and a plasmid with gene *recAEc* (pT420) were constructed in K.L. Knight’s laboratory [76]. Genes *recXEc* or *recXNg* were cloned in pT420 and in pD112R along with the expressed *recA* genes already present. Thus, pEAW958 is *recXNg* with an araBAD promoter in pD112R; pEAW959 is *recXNg* with an araBAD promoter in pT420(wt); pEAW858 is *recXEc* with an araBAD promoter in pD112R; and pEAW847 is *recXEc* with an araBAD promoter in pT420(wt). MG1655 with RecA D112R expressed on the chromosome at the normal r*ecA* locus is EAW166.

### Proteins

The wild type *E*. *coli* RecA, RecA D112R, RecX [38, 77] and SSB [27] proteins were purified as previously described. Their concentrations were determined using native extinction coefficients: ε_280_ = 2.23 x 10^4^ M^-1^ cm^-1^ for all RecA protein variants[78], and ε_280_ = 2.38 x 10^4^ M^-1^ cm^-1^ for SSB protein [79]. The concentration of *E*. *coli* RecX was determined from the absorbance at 280 nm using the native extinction coefficient 2.57 × 10^4^ m^−1^ cm^−1^. The *N*. *gonorrhoeae* RecX protein was purified as described [53]. Antibodies raised against the purified RecX protein were from "Genetel Lab" (Мadison, Wisconsin, USA).

### ssDNA dependent ATP hydrolysis (ATPase) Assays

A coupled enzyme, spectrophotomeric assay [80, 81] was used to measure RecA-mediated ATP hydrolysis. The ADP generated by hydrolysis was converted back to ATP by a regeneration system of pyruvate kinase and phosphoenolpyruvate (PEP). The resultant pyruvate was converted to lactate by lactate dehydrogenase using NADH as a reducing agent. The conversion of NADH to NAD^+^ was monitored as a decrease in absorbance at 380 nm. The amount of ATP hydrolyzed over time was calculated using the NADH extinction coefficient ε_380_ = 1.21 mM^-1^cm^-1^. The assays were carried out in a Varian Cary 300 dual beam spectrometer, with a temperature controller and a 12-position cell changer. The path length was 0.5 cm or 1 cm, the band pass was 2 nm. All ssDNA dependent ATPase assays contained a reaction solution of 25 mM Tris.OAc (pH 7.5, 80% cation), 10 mM MgOAc (except where noted), 3 mM potassium glutamate, 5% w/v glycerol, 1 mM dithiothreitol (DTT), 3 mM PEP, 30 U/ml pyruvate kinase, 30 U/ml lactate dehydrogenase, 4.5 mM NADH and 5 μM M13mp18 cssDNA. The wild-type RecA, RecAD112R (3 μM) were preincubated with ATP and 5 μm M13mp8 circular ssDNA for 5 min. SSB protein (0.5 μM) was then added, followed by another 5-min incubation. The time point of the RecX addition is shown by arrow (5 min). For reactions involving ATPase during DNA strand exchange, linear duplex M13mp18 DNA (10 μM) was added at the indicated time. Where Ec or NgRecX proteins were added, The final concentration is indicated in the figure and legend.

### Cell Culture

Each day, each of multiple stationary phase cultures grown overnight were diluted 50X and again grown to stationary phase in L-broth at 37°C (5–6 cell generations). A sample was taken at mid-log phase and used to measure conjugation proficiency. Cells were again diluted 50X into fresh L-broth. A sample was again taken at mid-log phase for the conjugation assay, and the culture was then allowed to grow overnight. The procedure was repeated for 6 days, or a total of approximately 70 cell generations.

### UV radiation sensitivity

Cells (EAW 166 (RecA D112R on the chromosome) and MG1655) were grown, serially diluted, and 100 μl of appropriate dilutions were spread onto LB plates. Dilutions for samples/treatments were empirically determined. The plates were then exposed to UV in a calibrated Spectrolinker XL-1000 UV crosslinker (Spectronics Corp) to the dose indicated. After incubating at 37°C overnight, the colonies were counted and divided by the dilution factor to get cfu/ml. For percent survival, colony counts on the treated plates were divided by the counts on untreated plates.

**Conjugation** was carried out essentially as described [82]. Both Hfr and F^-^ strains were grown, crossed and selected for recombinants at 37°C in mineral salts 56/2 medium supplied with all necessary growth factors at pH 7.5. The *recA* genes were not induced by IPTG but *recX* genes were induced by indicated concentration of arabinose. The ratio between donors and recipients in the mating mixture was 1:10, 2–4 x10^7^ donors and 2–4 x 10^8^ recipients per 1 ml. The yields of Ara^+^Str^r^ recombinants in all independent crosses were 0.1–7.8% relative to donors.

**FRE value calculations** were carried out as described [82]. During mating, the donor Hfr KL227 transfers markers into recipients in the order *leu*^*+*^, *ara*^*+*^ and *thr*^*+*^. Recombination exchanges in this region of the *E*. *coli* map are adequately described mathematically by the Haldane formula [82]. In the slightly rearranged form, λ = 2*l*/ln(2μ-1), this formula relates the average distance between two neighboring genetic exchanges (in minutes) to the linkage of selected and unselected markers (μ), and the distance (*l*), in minutes between markers on the *E*. *coli* map. The distance, ***l***, in minutes between the *thr* (0.05) and *leu* (1.75) markers is 1.7, and between the *ara* (1.47) and *leu* (1.75) markers is 0.28. Donor KL227 transfers *leu*^*+*^ and *thr*^*+*^ as a proximal and distal marker, respectively. The frequency of recombinational exchanges is expressed as FRE, the average number of exchanges per one *E*. *coli* genome equivalent (100 minutes), and thus equals 100/λ. For wild type *E*. *coli*, FRE = 5.0 [82]. In this study, we are particularly interested in changes in FRE, using the wild type *E*. *coli* values as a benchmark called FRE_2_. The ΔFRE is thus FRE_1_/FRE_2_, where FRE_1_ is simply the FRE measured for the cross under investigation. ΔFRE can also be calculated by use of the formula: ΔFRE = ln(2μ_1_−1)/ln(2μ_2_−1), where μ_1_ is the linkage observed with either a modified RecA or the wild type RecA under particular conditions of HR, and μ_2_ is the linkage observed with wild type EcRecA under standard conditions [83].

Alterations in FRE (ΔFRE) promoted by the *RecA D112R* and *RecXEc* or *RecXGc* genes or by the *EcRecA* and *RecXEc* or *RecXGc* genes relative to the FRE value promoted by the wild type *EcRecA* gene were calculated using the following formula: ΔFRE = ln(2μ_1_−1)/(2 μ_2_−1), where μ_1_ is the linkage of selected *ara*^*+*^ and unselected *leu*^*+*^ markers in a cross using wild type *E*. *coli* strain AB1157 and μ_2_ is the similar linkage in the cross being analyzed. Calculations of uncertainty of relative FRE values were determined as deviations from the average values by making use of the program Excel-97 with formula [= 2*STDEV] and by inputting the values from independent repeats of three experiments.

### Cell competition assays

Wild type cells and cells expressing the D112R variant of RecA protein at the normal *recA* chromosomal locus, were modified to carry a neutral Ara^−^mutation (which confers a red color on colonies when grown on tetrazolium arabinose (TA) indicator plates) to permit color based scoring of mixed populations [84]. Cells from a freezer stock of each strain were streaked on LB plates [85] and incubated at 37° C. After growth overnight, competition cultures were started by inoculating 3 mL fresh LB broth with an isolated colony of competition Ara+ or Ara^−^strains in triplicate and grown overnight at 37° C with shaking. Equal amounts of strains to be compared were mixed in triplicate. A sample of the mixture was taken, diluted by a factor of 10^−6^, and plated on tetrazolium arabinose indicator plates. Then, 3 mL fresh LB broth was inoculated with 30 μL of each mixture, and grown overnight. The plating, inoculation, and growth cycle was repeated two more times. White and red colonies were counted on plates containing 40–300 colonies, and the % of cells expressing mutant RecA proteins was determined. Normally for counting colonies, plates with fewer than 20 colonies of either competitor are excluded to reduce the effect of outliers caused by low counts [86]. However, at the 48 hour time point, because the differences in fitness is so great between wt recA and recA D112R, it was impossible to retrieve at least 40 colonies of the recA D112R in a range that could also be used to calculate the density of the wt recA strains.

### Visualizing intracellular RecX amounts by Western blot

*E*. *coli* cells were grown up to mid-log phase in LB medium at 37°C. A cell pellet containing 5 x10^7^ cells was lysed by boiling with sodium dodecyl sulfate, electrophoresed through sodium dodecyl sulfate-10% polyacrylamidе gels. The RecA or RecA D112R amounts were detected by immunoblotting using polyclonal chicken antibodies to RecA protein from “Genetel Lab” (Madison, Wisconsin, USA) in a standard procedure described earlier [87]. Primary antibody binding was visualized with secondary antibodies coupled to horseradish peroxydase (Genetel Lab).

### Strand Exchange reaction

Three-strand exchange reactions were carried out in 25 mm Tris-OAc buffer (80% cation), 1 mm dithiothreitol, 5% (w/v) glycerol, 3 mM potassium glutamate, 2 mM ATP and 10 mM of Mg(OAc)_2_. Reactions also contained an ATP regeneration system of 30 units/ml pyruvate kinase and 5 mM phosphoenolpyruvate. The spectrophotometric conditions were the same as for ssDNA dependent ATP hydrolysis. All incubations are carried out at 37 °C. The wild-type RecA, RecAD112R (3 μM) were preincubated with ATP and 5 μm M13mp8 circular ssDNA for 5 min. SSB protein (0.5 μM) was then added, followed by another 5-min incubation. The reactions were initiated by the addition of M13mp8 linear dsDNA to 10 μM. The time point of dsDNA addition is shown by arrow. DsDNA was added at 5 min on the graph. The RecX was added at 30 min after dsDNA or 35 min on the graph (shown by arrow). On the some graph RecX was added at 10 min after dsDNA.

### Growth Curves

LB media (25 ml) was inoculated with 0.25 ml of a fresh (to avoid de-evolution) culture of a strain to be tested and grown for the indicated times at 37°C with shaking at 200 rpm. The concentration of arabinose was 0.00002% for RecX induction but IPTG was not added. Culture aliquots were removed every hour, and growth was monitored by absorption of the sample at a wavelength of 600 nm, using a Cary 100 spectrophotometer. Solutions were diluted as needed to bring the absorption into the linear range of the spectrophotometer.

### Comparative Genome Sequencing (microarray analysis)

DNA samples were analyzed to detect genomic alterations using the Comparative Genome Sequencing Service provided by NimbleGen Systems, Inc. as described earlier [88].

### Direct DNA sequencing

Standard Sanger sequencing was carried out at the University of Wisconsin Biotechnology Center, after PCR amplification of the target genomic DNA sequence.

## Results

### Expression of RecA D112R confers a growth disadvantage

**Fig 1:**
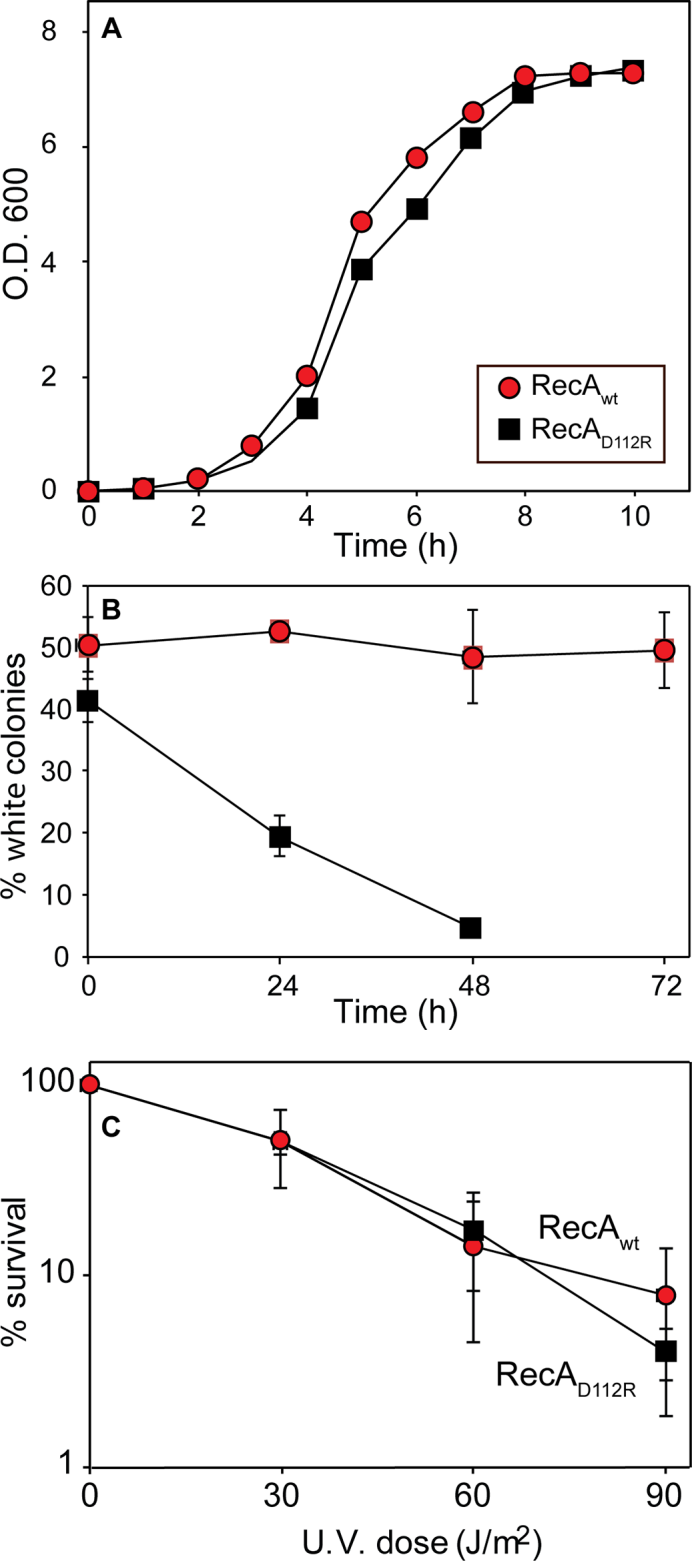
Growth disadvantage conferred by RecA D112R. (A) Growth curves for *E*. *coli* cells expressing either the wild type RecA protein (red circles) from plasmid pRecA or the RecA D112R variant (black squares) from plasmid pRecA[D112R]. (B) Cell growth competition assays. Assays were carried out as described in Methods. The top trial (red circles) shows a competition between two wild type cultures, one of which carries the Ara^−^mutation. The lower one shows a competition between wild type cells and cells expressing RecA D112R at the normal chromosomal *recA* locus. The *ara*^*−*^mutation conferring a red color to colonies is present in the cells expressing wild type RecA in this experiment. Colony counts revealing the % of cells expressing the mutant RecA protein are plotted as a function of the daily growth cycle of the experiment. (C) Resistance to ultraviolet light. Experiments were carried out as described in Materials and Methods. The strains compared are MG1655 and EAW166 (RecA D112R expressed on the chromosome at the normal *recA* locus).

The RecA D112R variant provides a substantial increase in function with respect to conjugational recombination, utilizing two different conjugation assays [71, 72]. To explore the deleterious consequences of this increase in functionality, we first examined cellular growth rates. As shown in Fig 1A, cells expressing the RecA D112R variant grow somewhat more slowly than do cells expressing the wild type RecA protein. The difference appears small, but it proved highly reproducible over the course of 4 trials. To provide a more sensitive measurement of any positive or deleterious effects of the *recA* mutations on cell growth and survival, we carried out direct competition assays between strains expressing wild type or the D112R *recA* protein, each present at the same normal chromosomal for *recA* [84]. Wild type cells were modified to carry a neutral Ara^−^mutation (which confers a red color on colonies when grown on tetrazolium arabinose (TA) indicator plates) to permit color based scoring of mixed populations [84]. Overnight cultures of cells expressing the D112R *recA* variant were mixed in a 50/50 ratio with isogenic wild type cells, with one strain carrying the Ara^−^mutation. The ratio of wild type to mutant strains was then followed over the course of several days, with stationary phase cultures diluted and grown out each day as described in Methods. Results are presented in Fig 1B. Based on earlier work, the Ara^−^mutation itself should not affect growth rates [84, 89]. In all experiments, the strain expressing RecA D112R was largely eliminated from the mixed population in less than three days. We conclude that expression of RecA D112R, even from the normal *recA* locus, confers a significant growth disadvantage. We have previously noted a similar growth disadvantage in *E*. *coli* cells expressing RecA proteins with enhanced function [72]. Here, we carry out the first experiments to determine how the cell responds to this problem.

We also tested the effect of RecA D112R on cellular sensitivity to ultraviolet radiation (Fig 1C. With the RecA variant expressed from the normal chromosomal locus, there was little evident effect.

### Growth of cells expressing RecA D112R leads to de-evolution of the hyperrec phenotype

In this study and as described in Methods, we are reporting the frequency of recombinational exchanges (FRE) during the conjugational cross, defined as the average number of exchanges per one *E*. *coli* genome equivalent (100 minutes). For wild type *E*. *coli*, FRE = 5.0 [82]. In previous work, we demonstrated that FRE is increased by over 50 fold when RecA D112R replaces the wild type RecA protein in *E*. *coli*. Cells expressing RecA D112R thus exhibit a hyperrec phenotype [71]. We wished to determine whether the hyperrec phenotype was stable.

**Fig 2:**
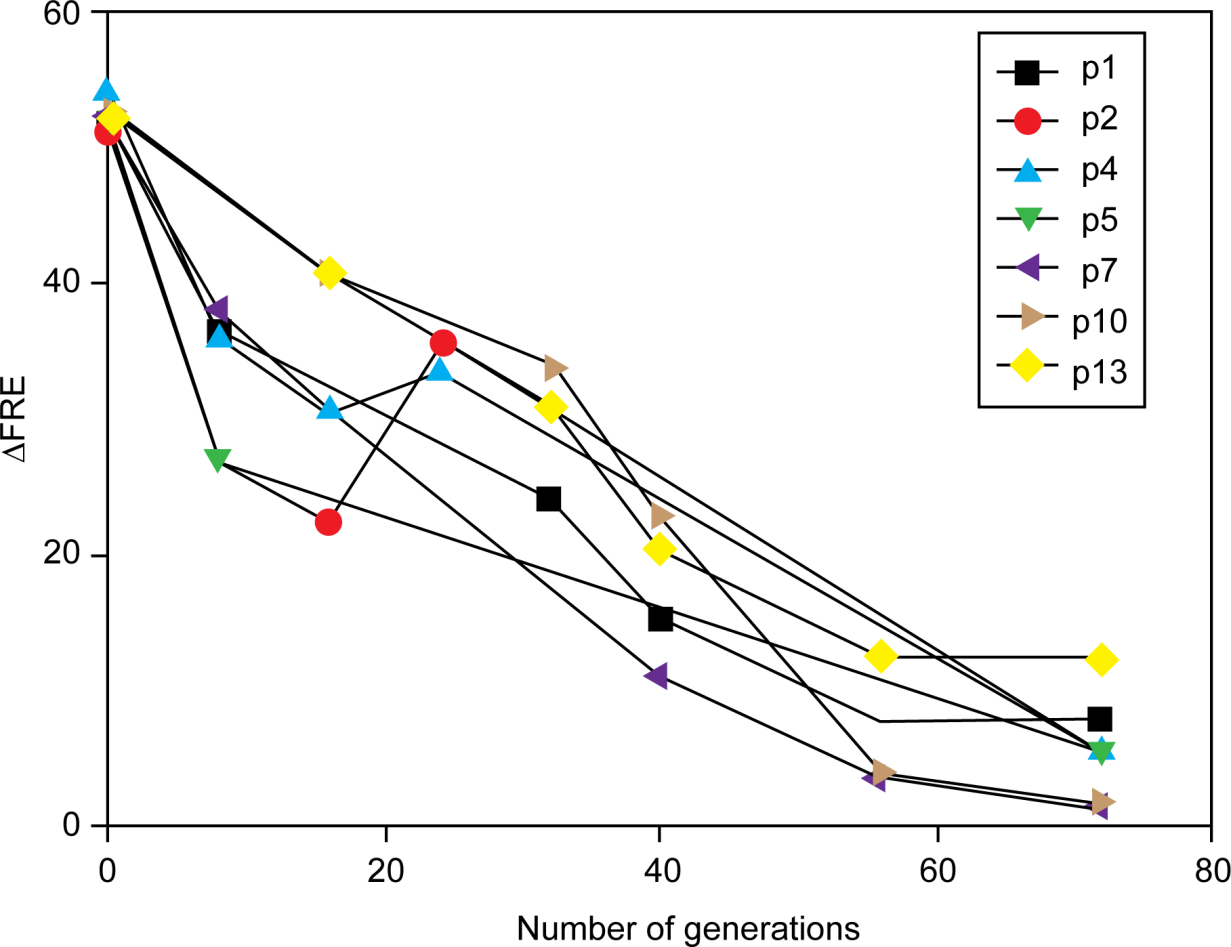
Loss of hyperrec phenotype in cells expressing RecA D112R upon continuous culture. Multiple cultures of strain JС10289, expressing RecA D112R protein from plasmid pRecA[D112R] were grown as described in Materials and Methods for 70 generations. ΔFRE was determined for each culture at the indicated times, and is the ratio of the FRE value measured for JC10289 with pRecA[D112R] to the FRE value for JC10289 expressing wild type RecA protein.

Continuous cycles of growth and dilution of a strain expressing RecA D112R results in a decline in the measured ΔFRE value (the ratio of FRE values of the strain being measured relative to that observed for wild type cells) (Fig 2). Over 70 cell generations, a marked decline in ΔFRE was seen for all 16 cultures of *ΔrecA* strain JC10289, with a plasmid expressing RecA D112R (pRecA [D112R]). The molecular basis for the decline generally fell into three categories: (a) a deletion in pRecA [D112R] that reduced expression of the RecA variant, (b) a mutation in the host chromosome that impacted RecA D112R expression, or (c) a combination of the two.

The three effects can be seen in the results tabulated in Table 1. The final ΔFRE for the 16 separate cultures are listed in the first data column. In each case, this measure of conjugation function has been reduced from over 50 to a range of 1.2–20.3.

**Table 1:** Loss of the hyperrec phenotype in strains expressing RecA D112R protein. The second column documents ΔFRE, the relative increase in the frequency of genetic exchange per chromosomal DNA unit length (ΔFRE is a ratio of the FRE observed with the indicated strain divided by FRE for comparable cells expressing the wild type RecA protein), after continuous culture for 70 cell generations. The third column shows results obtained after the plasmids from the cultured strains were isolated and used to again transform strain JC10289. Four of the plasmids (from cultures 8, 11, 12, and 13) still produce an elevated ΔFRE. In the final column, the strains were cured of their resident plasmid, and re-transformed with pRecA D112R. The lower ΔFRE scores in some of these strains reflect the effects of chromosomal mutations.

Strain №	ΔFRE	ΔFRE JС10289/ pRecA[D112R]_Mod_	ΔFREJC10289_Mod_/ pRecA[D112R]
**1**	**7.8**	**1.3**	**-**
**2**	**5.7**	**1.3**	**-**
**3**	**2.2**	**1.7**	**14.1**
**4**	**5.6**	**1.3**	**20.8**
**5**	**5.4**	**1.3**	**-**
**6**	**1.2**	**2.4**	**13.8**
**7**	**1.5**	**3.0**	**12.4**
**8**	**10.8**	**58.8**	**7.2**
**9**	**12.0**	**2.0**	**-**
**10**	**1.7**	**1.7**	**31.3**
**11**	**13.4**	**50.0**	**15.3**
**12**	**20.3**	**65.4**	**4.1**
**13**	**12,4**	**68.7**	**13.5**
**14**	**3.0**	**0.7**	**-**
**15**	**5.6**	**2.2**	**-**
**16**	**6.0**	**3.2**	**-**

**Fig 3:**
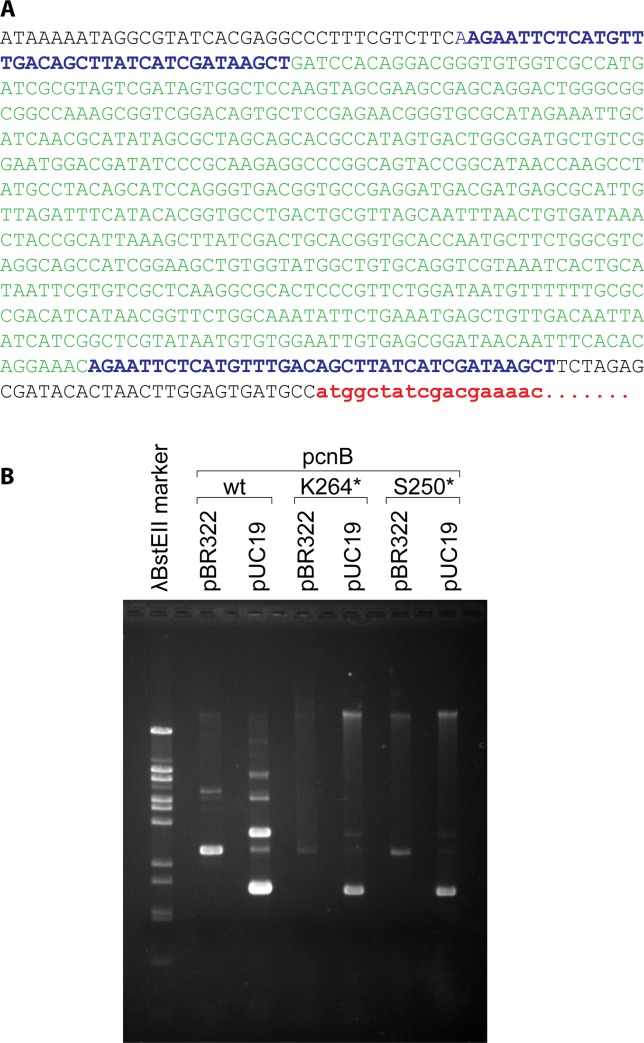
Plasmid and genomic changes detected in de-evolution trials. (A) The 633 base pair deletion (green) was detected in the promoter upstream of the gene expressing RecA D112R as described in the text. The 37 bp repeats that appear to facilitate the deletion are shown in blue. The beginning of the gene expressing RecA D112R is shown in red. (B) Effect of *pcnB* mutations on the maintenance of plasmids pBR322 and pUC19. Cells with or without the indicated *pcnB* mutations were grown and harvested, and plasmid DNA was extracted. The plasmid DNA shown in each lane represents the amount isolated from 1.5 ml of stationary phase cells at OD_600_ = 6.6–6.9, except for MG1655 (WT RecA) containing pUC19, which represents 3 ml of stationary phase cells at OD_600_ = 3.0. All OD_600_ measurements were carried out after 1/10 dilution of the cultures listed so that readings remained in the linear range of the spectrophotometer. The very high copy number of pUC19 in strain MG1655 may limit cell growth. The * indicates that the mutation represents the introduction of a stop codon at the residue position K264 or S250 indicated. The experiment was repeated twice with consistent results.

When the plasmids derived from the culture endpoints were isolated and used to transform strain JC10289 again, the plasmid preps produced low ΔFRE values indicative of loss of the hyperrec phenotype in 12 of 16 cultures. This indicated that the genetic alteration that produced the loss of phenotype was present on the plasmid in those 12 cases. The entire protocol featuring continuous cycles of growth and dilution of the strain expressing RecA D112R was repeated for an additional 6 cultures, and a plasmid alteration was again detected in 3 of them. Sequencing of the plasmids derived from the 3 new cultures revealed a common 633 bp deletion in the promoter region for the *recA D112R* gene, produced by apparent recombination between two 37 bp direct repeats (Fig 3A).

In four of the cases highlighted in Fig 2 and Table 1, (cultures 8, 11, 12, and 13), the isolated plasmids still produced a high ΔFRE value, suggesting that the mutations affecting the loss of the hyperrec phenotype were chromosomal. Strains 8 and 12 were subjected to microarray analysis to identify mutations relative to the *E*. *coli* genome reference sequence [90], and chromosomal mutations were confirmed by direct sequencing. Most of the mutations detected were present in the founder strain JC10289 used as the host for these experiments, as well as the de-evolved strains. Notably, in both strains 8 and 12 there was a mutation that may have inactivated the *pcnB* gene, important for the maintenance of colE1-based plasmids [91–94]. The two different *pcnB* mutations (which introduce stop codons at either codon 250 or 264) were moved into an otherwise wild type background, and the resulting strains were tested for maintenance of plasmids pUC19 and pBR322, which share the replication origin of pRecA [D112R].

As shown in Fig 3B, the plasmids pUC19 and pBR322 appeared to be maintained at significantly lower levels in cells with either of the *pcnB* mutations. This experiment was repeated three times with identical results. Data ancillary to Fig 3B is presented in Table 2. Colony forming units (CFU) generally scaled with the optical density of the overnight cultures. Those O.D.s were similar except for MG1655 hosting pUC19, which was less than half of the density and less than half of the CFUs in an identical culture volume. Isolation of plasmid DNA as in Fig 3B produced apparent plasmid DNA levels (as measured by optical density at 260 nm) that are reported in Table 2, although this measurement includes RNA contamination. The reduction in overall nucleic acid in strains with pUC19 when pcnB mutations are present are readily evident in this assay. The changes in pBR322 levels are not evident, possibly because of the presence of RNA or some other contaminant.

**Table 2:** Data reflecting plasmid levels in pcnB mutants.

strain	O/N OD^2^	CFU^3^	Plasmid^4^
MG1655/pBR322	6.15 ±0.35	244 ±44	71.4 ±11.5
pcnB K264*/pBR322^1^	6.71 ±0.27	183 ±22	58.8 ±27.8
pcnB S250*/pBR322	6.68 ±0.09	206 ±21	51.7 ±8.7
MG1655/pUC19	2.97 ±0.08	70 ±13	172.2 ±14.2
pcnB K264*/pUC19	6.67 ±0.29	200 ±16	78.2 ±14.6
pcnB S250*/pUC19	6.75 ±0.11	239 ±21	71.7 ±3.5

1. The * denotes a stop codon at the position indicated.

2. Total optical density of an overnight culture measured (after dilution of 1/20) at 600 nM (n = 4).

3. Colony forming units found after spreading 50 μl of a 10^−6^ dilution of the overnight culture (n = 3).

4. Plasmid DNA (plus any contaminating nucleic acid; in ng/μl) isolated from 3 ml of an overnight culture as determined by optical density at 260 nm.

**Fig 4:**
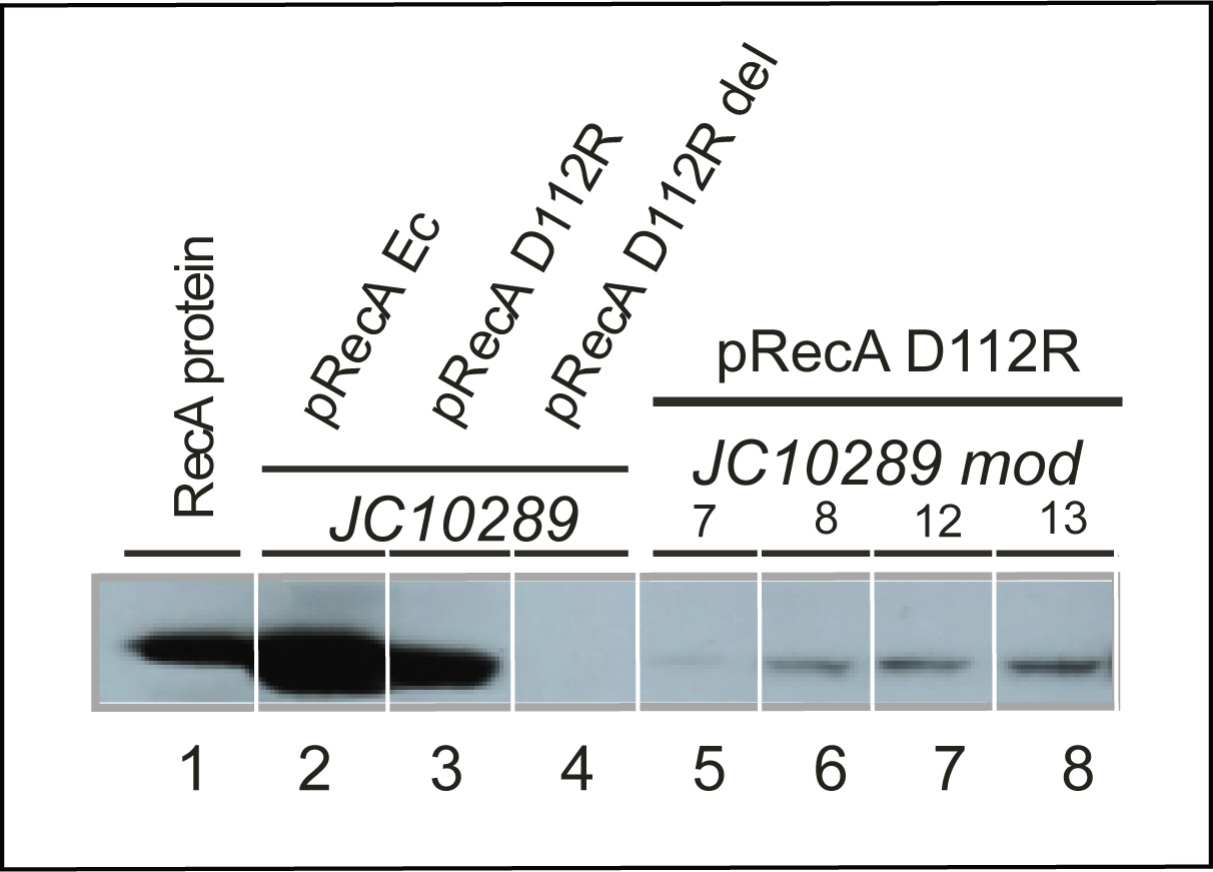
RecA protein expression in cells that have lost the hyperrec phenotype. Intracellular RecA protein was measured by Western blotting as described in Materials and Methods. Each lane reflects the RecA protein content of 5 X 10^7^ cells. Lane 1 is a marker. Lanes 2 and 3 represent expression of either EcRecA (lane 2) or RecA D112R (lane 3) from their respective plasmids controlled by the *tac* promoter in the absence of induction. Lane 4 shows an empty vector control. Lanes 5–8 show results from de-evolved strains 7, 8, 12, and 13 respectively as labeled.

In a separate experiment, the levels of expression of RecA D112R were measured and were substantially reduced in strains 7, 8, 12, and 13 (Fig 4). Intracellular RecA D112R protein levels were reduced dramatically in all four strains. The *pcnB* mutations provide one, but possibly not the only explanation for the reduction in RecA D112R expression.

Independently, we cured nine of the cultured strains of their resident plasmids, and transformed them with the original plasmid expressing RecA D112R. The results are tabulated in the final data column of Table 1. In no case did this produce a strain that exhibited the ΔFRE level of the original strain. In strains 8, 11, 12, and 13, the ΔFRE values were comparable to those seen after 70 generations of growth in the original experiment, indicating that background chromosomal mutations were dominant in diminishing the hyperrec phenotype. In strains 3, 4, 6, 7, and 10, the measured ΔFRE increased, but not to the ΔFRE level of 50+ observed in the original strain. The results suggest the presence of background alterations in the chromosome that moderate the effects of RecA D112R expression and/or function, in addition to alterations on the plasmid.

### The growth deficiency conferred by RecA D112R expression reflects persistent binding to the *E*. *coli* chromosome

In vitro, the RecA D112R protein rapidly displaces SSB to nucleate on ssDNA, and also binds to duplex DNA more readily than the wild type RecA protein [71]. These properties could produce more extensive binding to chromosomal DNA in *E*. *coli*, and lead to inhibition of normal chromosomal DNA replication. To explore this possibility, we utilized the RecX protein in the attempt to disrupt the RecA filaments and restore normal growth rates on the *E*. *coli* cells. The *E*. *coli* RecX protein (EcRecX) works primarily by binding to the 3'-proximal end of the RecA filament, blocking RecA filament extension. Net dissociation of the filament then occurs at the 5'-proximal end [52]. The RecX protein derived from the bacterium *Neisseria gonorrhoeae* (NgRecX) has a more robust activity, creating internal filament discontinuities that trigger more rapid filament disassembly [53]. Both proteins were tested with RecA D112R.

**Fig 5:**
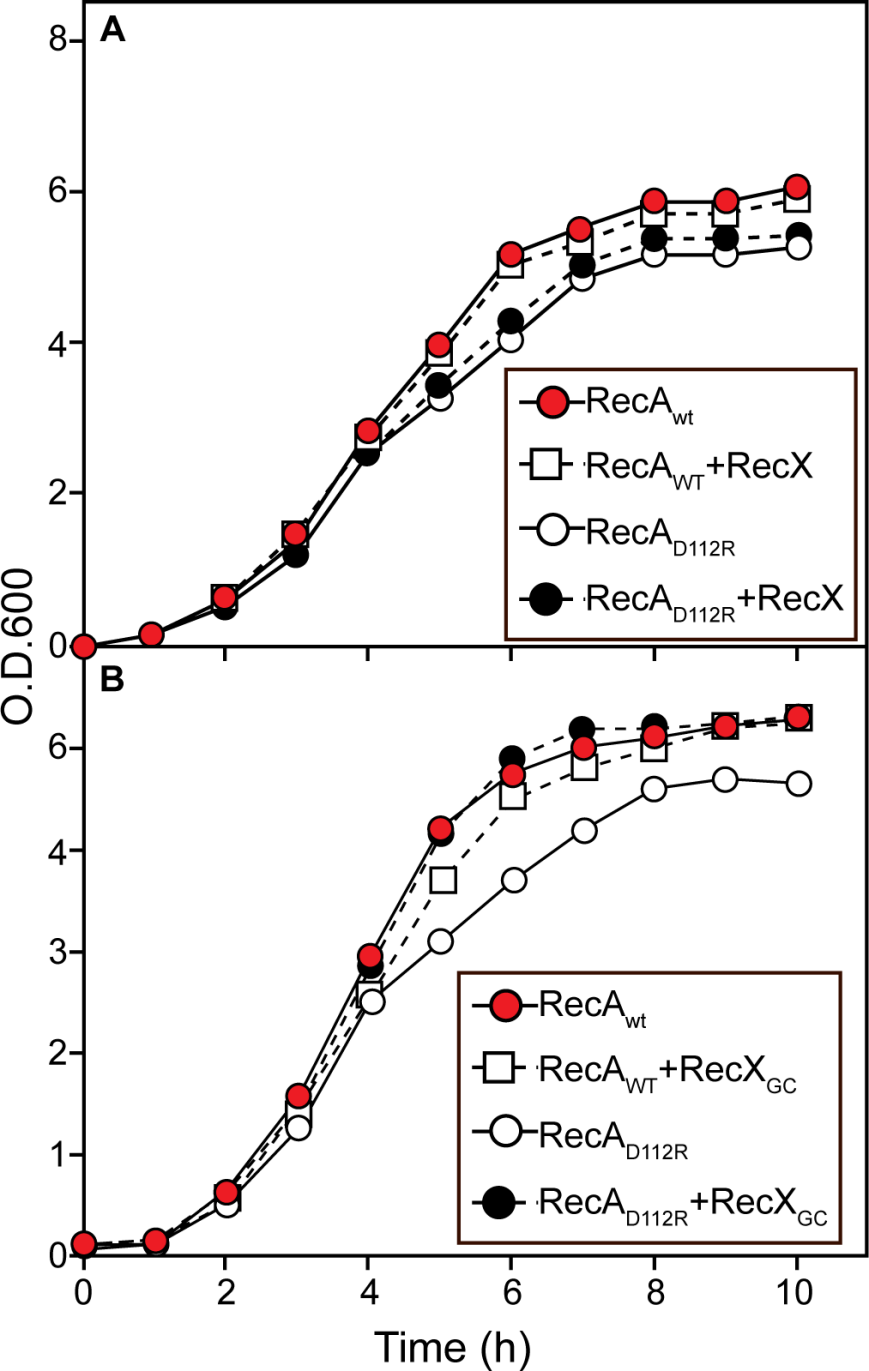
Effects of RecX proteins on the growth curves of cells expressing wild type RecA protein or RecA D112R protein. Cells containing pRecA Ec or pRecA [D112R] were grown in LB with Ampicillin 100mkg/ml. Where indicated, the cells contained plasmid pEAW847, expressing the RecX and RecA proteins from *E*.*coli*, pEAW858 –RecX and RecA D112R proteins from *E*. *coli*, pEAW959 –RecA from *E*.*coli* and RecX_NG_, or pEAW958 –RecA D112R and RecX_NG_.

As shown in Fig 5A, the expression or overexpression of EcRecX has little effect on the growth rates of *E*. *coli* cells expressing either wild type RecA or RecA D112R. In contrast, expression of the more robust NgRecX from a plasmid restores normal growth rates to the cells (Fig 5B). These experiments were repeated 3 times with consistent results.

**Fig 6:**
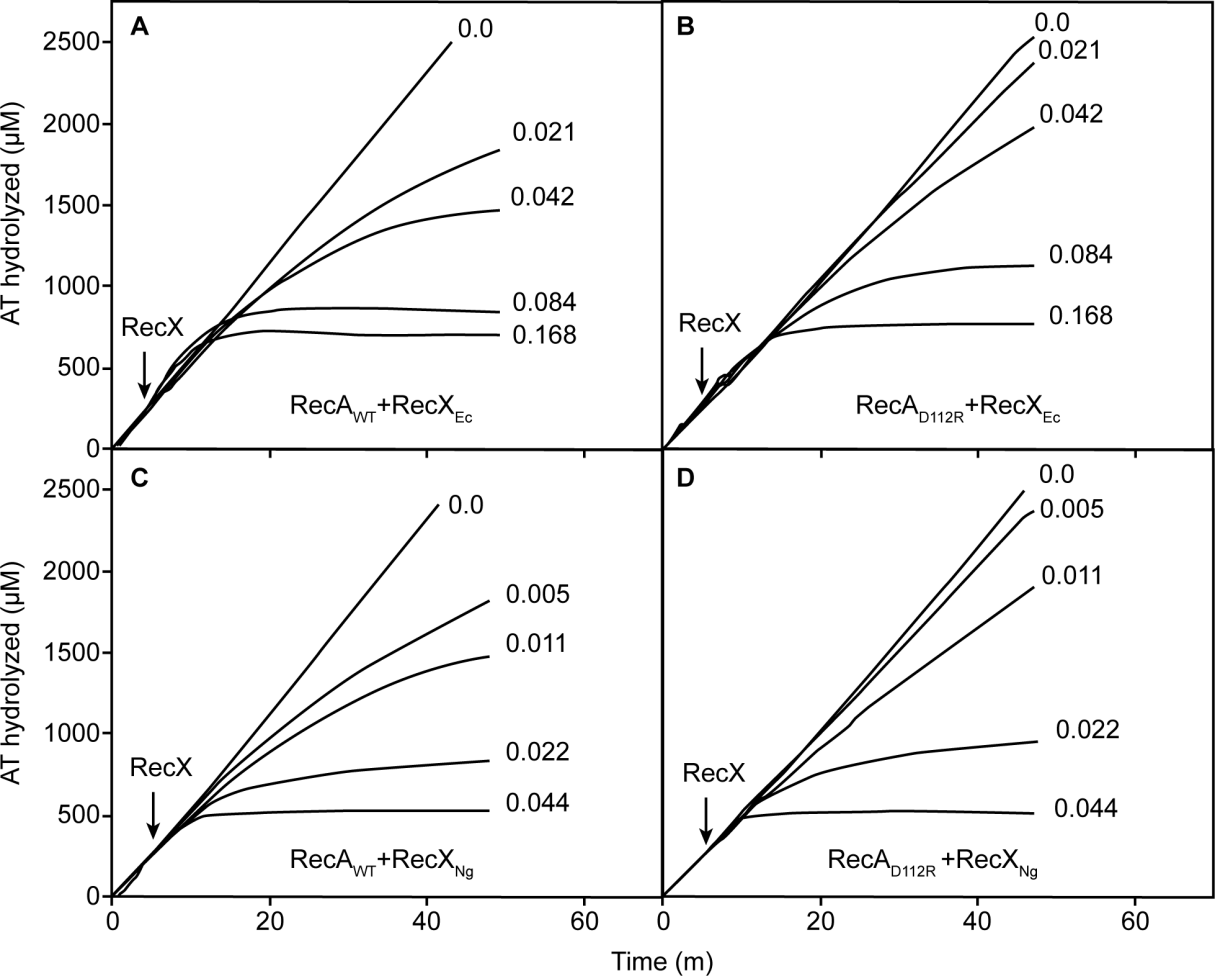
Inhibition of RecA-mediated and DNA-dependent ATP hydrolysis by RecX proteins. ATPase reactions were carried out and monitored as described in Materials and Methods, with 5 μM RecA or RecA D112R protein as indicated and RecX protein (from *E*. *coli*) or RecX_NG_, as indicated. RecX proteins were added at the time indicated to a final concentrations indicated for each line (in μM).

**Fig 7:**
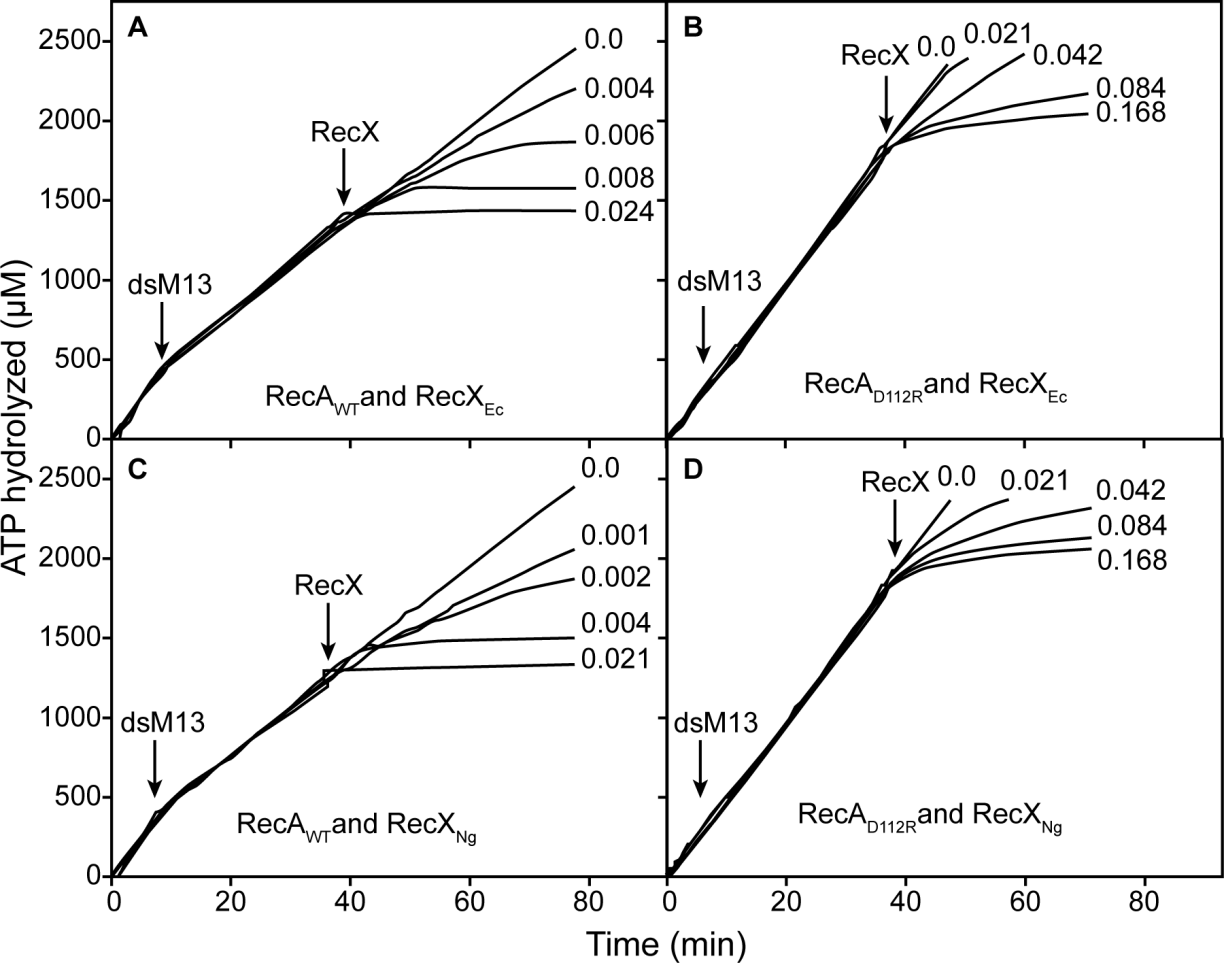
Inhibition of RecA-mediated ATP hydrolysis by RecX proteins during DNA strand exchange. ATPase reactions were carried out and monitored as described in Materials and Methods, with 5 μM RecA or RecA D112R protein as indicated and RecX protein (from *E*. *coli*) or RecX_NG_, as indicated. Duplex linear M13mp18 DNA was added at the time indicated by “dsM13”. RecX proteins were added at the time indicated to a final concentrations indicated for each line (in μM).

The differential effects of the two RecX proteins are readily seen in vitro (Fig 6). To produce a given level of inhibition of the RecA or RecA D112R ATPase, about 4 fold less NgRecX was required than was EcRecX (Fig 6). About 2 fold more of either RecX protein is needed for the RecA D112R protein than is required for wild type RecA. The resistance of RecA D112R protein to RecX increases during DNA strand exchange. If the RecX proteins are added when RecA is actively promoting DNA strand exchange, the amount of either RecX needed to obtain a given level of inhibition of RecA D112R was about 20–40 fold greater than seen for the wild type RecA protein (Fig 7). Again, NgRecX was more effective than EcRecX, by a factor of at least 2.

The effects of the two RecX proteins were determined with more resolution by measuring their effects on FRE in the various constructs. The genes encoding the *E*. *coli* RecX protein or the Neisseria RecX (RecX_NG_) were expressed from the arabinose promoter. The RecA proteins were expressed from the *tac* promoter, but no IPTG inducer for *tac* was added. The level of the RecX proteins was varied by increasing the concentration of arabinose added to the media, and FRE levels were monitored (Table 3). As expression of the wild type RecX protein from *E*. *coli* was increased to high levels by arabinose addition, FRE levels declined in cells expressing either the wild type RecA protein or RecA D112R. However, the decline was greater when RecX_NG_ was expressed. In general, the RecA D112R mutant protein was significantly less sensitive to either RecX protein than was the wild type RecA protein.

**Table 3:** Effect of RecX protein expression on measured FRE levels in strains expressing RecA or RecA D112R proteins. The strain used is JC10289 (AB1157 ΔrecA) engineered with a deletion of the araBAD operon as described in Materials and Methods. Plasmids were constructed to express both the indicated RecA (from the *tac* promoter, uninduced) and the indicated RecX (from the *ara* promoter, with arabinose added as an inducer as indicated) proteins.

	RecX *Ec*		RecX *Gc*		None	
RecA	wt	D112R	wt	D112R	wt	D112R
0.0% ara	2.4%* 0.935±0.016(600)**FRE = 24.8***	5.3% 0.548±0.017(600) **FRE = 416.7**	0.67% 0.943±0.005(900)**FRE = 21.7**	0.26% 0.554±0.032(900)**FRE = 400.0**	4.5% 0.939±0.007(800)**FRE = 23.2**	0.82% 0.560±0.006(600)**FRE = 384.6**
0.01% ara	4.6% 0.928±0.011(1200) **FRE = 27.8**	4.1% 0.642±0.020(900) **FRE = 227.3**	0.68% 0.960±0.003(1200)**FRE = 14.8**	0.85% 0.763±0.073(600)**FRE = 114.7**		
0.1% ara	5.5% 0.981±0.009(900) **FRE = 6.9**	7.8% 0.843±0.032(900) **FRE = 67.3**	0.48% 0.993±0.007(500)**FRE = 2.5**	0.61% 0.900±0.020(600)**FRE = 41.1**		
1% ara	4.5% 0.986±0.008(1200) **FRE = 5.0**	4.3% 0.944±0.017(1220)**FRE = 21.3**	011% 0.996±0.004(900)**FRE = 1.5**	0.09% 0.984±0.008(700)**FRE = 5.8**		

* Yield of Thr+Strr recombinants (% to donors)

**Linkage (m) between selected *thr* + and unselected *leu* + markers

***Frequency of recombination exchanges per DNA unit length (FRE)

## Discussion

The hyperrec phenotype conferred by expression of the RecA D112R variant confers a readily demonstrable cell growth deficiency. Whereas the difference in the rate of growth appears small, the defect provides a substantial selective disadvantage. Upon continuous culturing, rapid loss of the hyperrec phenotype occurs producing mutations that decrease the expression of RecA D112R. We conclude that an enhancement of RecA function alters an existing balance between different processes in DNA metabolism, giving rise to a strong selection pressure to restore that balance.

The growth deficiency most likely reflects an increase in binding of RecA D112R to the bacterial chromosome, impeding other processes in DNA metabolism. The growth deficiency is alleviated by expression of a potent RecA inhibitor, the NgRecX protein, which facilitates a robust removal of RecA protein from DNA. The NgRecX protein is also more effective in the inhibition of RecA D112R functions in vitro. The NgRecX protein provides an artificial path to restoring that balance. The only known function of RecX protein is the removal of RecA protein from the DNA, and the NgRecX protein has a particularly robust capacity to carry out that function [53]. The capacity of NgRecX to eliminate the growth deficiency in cells expressing RecA D112R implies that this RecA variant simply binds to chromosomal DNA too persistently, impeding other processes in DNA metabolism. In the absence of NgRecX protein provided artificially, the balance is restored via adaptations that reduce RecA D112R expression. Functional enhancement of RecA is not well-tolerated by *Escherichia coli*.

It has become clear that RecA protein properties can limit replication and cell growth rates [72, 95]. The present work reinforces this conclusion, but also provides a first look at how a bacterial cell responds to the limitations. Even the modest changes in cell growth rate imposed by a RecA filament that is resistant to removal from DNA by RecX create a selective pressure sufficient to select for adaptive alterations in the expression of the responsible RecA variant. In the cases explored here, the cells adapted by limiting expression of the RecA variant, either by alterations to the RecA variant promoter or by chromosomal alterations in the *pcnB* gene that reduced plasmid copy number. We have not yet searched exhaustively for genomic changes that could permit cell adaptation to a RecA variant that binds to DNA too persistently.

It is conventional to assume that each cellular protein has evolved to optimize its molecular function. In the case of proteins involved in DNA metabolism, this is not necessarily the case. Instead, evolution has refined a balance between the different aspects of DNA metabolism that must all share the same chromosomal substrate. Thus, the RecA protein existing in each bacterial species is not optimized for the promotion of DNA pairing and strand exchange per se. Recombinase function can be altered in a variety of ways, increasing cellular resistance to ionizing radiation [96] or proficiency in conjugation [71, 72]. If RecA protein filament disassembly is slowed, cell growth rates are slowed [95]. It is possible to make a better recombinase, but increased functionality does not always result in enhanced fitness.

## Conclusion

All processes in DNA metabolism–replication, repair, recombination, transcription, packaging–share the same chromosomal DNA substrate. Conflicts are common and are mediated by a wide range of mechanisms honed by evolution. The RecA protein filament represents the largest contiguous structure assembled on a bacterial chromosome, a potentially substantial barrier to replication. RecA has not evolved to promote its DNA strand exchange reaction optimally. Instead, evolution has crafted a functional compromise appropriate to cellular requirements. Functional enhancements in RecA protein are possible that produce a hyperrec phenotype. However, they lead to a growth disadvantage and a substantial selection pressure to reverse the adverse effects. A RecA-based perturbation of the DNA metabolic balance creates a strong selective pressure that leads to rapid loss of the hyperrec phenotype. In the cases we document, this involves chromosomal or plasmid alterations that result in a reduction in the expression of the *recA* gene variant.
